# Identification of consistent QTL and candidate genes associated with seed traits in common bean by combining GWAS and RNA-Seq

**DOI:** 10.1007/s00122-024-04638-5

**Published:** 2024-05-27

**Authors:** Maria Jurado, Carmen García-Fernández, Ana Campa, Juan Jose Ferreira

**Affiliations:** Plant Genetic Group, Regional Service for Agrofood Research and Development (SERIDA), 33300 Villaviciosa, Asturias Spain

## Abstract

**Key message:**

**Association analysis, colocation study with previously reported QTL, and differential expression analyses allowed the identification of the consistent QTLs and main candidate genes controlling seed traits.**

**Abstract:**

Common beans show wide seed variations in shape, size, water uptake, and coat proportion. This study aimed to identify consistent genomic regions and candidate genes involved in the genetic control of seed traits by combining association and differential expression analyses. In total, 298 lines from the Spanish Diversity Panel were genotyped with 4,658 SNP and phenotyped for seven seed traits in three seasons. Thirty-eight significant SNP-trait associations were detected, which were grouped into 23 QTL genomic regions with 1,605 predicted genes. The positions of the five QTL regions associated with seed weight were consistent with previously reported QTL. HCPC analysis using the SNP that tagged these five QTL regions revealed three main clusters with significantly different seed weights. This analysis also separated groups that corresponded well with the two gene pools described: Andean and Mesoamerican. Expression analysis was performed on the seeds of the cultivar ‘Xana’ in three seed development stages, and 1,992 differentially expressed genes (DEGs) were detected, mainly when comparing the early and late seed development stages (1,934 DEGs). Overall, 91 DEGs related to cell growth, signaling pathways, and transcriptomic factors underlying these 23 QTL were identified. Twenty-two DEGs were located in the five QTL regions associated with seed weight, suggesting that they are the main set of candidate genes controlling this character. The results confirmed that seed weight is the sum of the effects of a complex network of loci, and contributed to the understanding of seed phenotype control.

**Supplementary Information:**

The online version contains supplementary material available at 10.1007/s00122-024-04638-5.

## Introduction

Common bean (*Phaseolus vulgaris* L.) is a diploid and self-pollinated species that is considered the most important legume crop for direct human consumption (FAO 2022). Bean crops are present worldwide, and depending on their genotype, they can be consumed as immature pods (snap beans or green beans) or seeds after rehydration (dry beans). Bean seeds are a valuable source of proteins, carbohydrates, dietary fiber, vitamins, minerals, and bioactive molecules as phenolic components (Hayat et al. 2014). In addition, bean crops provide benefits to the soil, have low carbon and water footprints, and integrate well into sustainable agricultural models (Uebersax et al. 2022).

In the common bean, two main gene pools were found in the analysis of variation in morpho-agronomic traits, seed size, isoenzymes, seed proteins such as phaseolin, and different types of DNA markers in both wild and cultivated populations (Gepts et al. 1986; Singh et al. 1991; Blair et al. 2009): Andean (A) and Mesoamerican (MA). Each gene pool was domesticated independently in parallel domestication events. These two gene pools have also been observed in the European germplasm, although cultivars showing different levels of introgression between both gene pools have also been detected (Santalla et al. 2002; Campa et al. 2018).

Bean seeds exhibit extensive phenotypic variation (e.g., Campa et al. 2018), which can be described by considering a combination of seed shape, seed size, seed coat color, and color distribution. Seed shape is recorded as seed dimensions (length, width, thickness, and area) and the ratios among them, whereas seed size is usually recorded as 100 seed weight. The seed phenotype is an important trait in domestication and is related to consumer acceptability and its potential use as precooking and canned food. Seed size is a yield-related trait, along with the number of pods per plant and the number of seeds per pod (White and González 1990). Seed phenotype is also an important trait in snap bean varieties, which are preferred by white seed color, elongated seed shape, and smaller seed size (Silbernagel et al. 1991). Other relevant characteristics of bean seeds are water absorption and coat proportion because of their relationship with cocking response and consumer acceptability (Berry et al. 2020). Water uptake during soaking has been correlated with cooking time, and there is a relationship between seed size and speed of water absorption (Vidak et al. 2022). The seed coat plays a significant role in the hard-to-cook process of bean hardening before and during storage (de León et al. 1989). The seed coat represents approximately 10% of the seed weight and shows high mineral content (for example, Fe, Ca, and Mg) and antioxidant capacity, as well as many anti-nutrients that affect mineral bioavailability (Blair et al. 2013). The proportion of seed coat is negatively correlated with seed hardness; seeds with a higher percentage of coat tend to have hard shells (Escribano et al. 1997).

Some studies have addressed the inheritance of the traits involved in seed phenotypes. Seed size, shape, water uptake, and coat proportion exhibit quantitative inheritance with moderate-to-high heritability (Moghaddam et al. 2016; Berry et al. 2020). Many quantitative trait loci (QTL) involved in the genetic control of these seed traits have been described (González et al. 2016; Murube et al. 2020), although some QTL for seed size and shape have been collocated in different backgrounds and studies (Murube et al. 2020). These studies were conducted in different environments and used biparental populations, which revealed variation between the involved parents. Genome-wide association studies (GWAS), in which variation is captured among a defined population, have also reported genomic regions associated with seed size, shape, and quality traits (Blair et al. 2009; Schmutz et al. 2014; Cichy et al. 2015; Moghaddam et al. 2016; Giordani et al. 2022; Amongi et al. 2023). All of these genetic studies have described major, minor, and epistatic QTL for seed traits across all 11 common bean chromosomes (Arriagada et al. 2022). However, most of the QTL involved in the inheritance of seed phenotype traits have not been validated in different backgrounds (genotypes and environments) or are not well delimited in the bean genome, an important feature before being used in plant breeding.

Concerning the candidate genes controlling seed morphological traits, Schmutz et al. (2014) suggested 15 candidate genes associated with seed weight in a Mesoamerican panel consisting of 280 genotypes, three of which were highlighted by (Moghaddam et al. 2016) for seed weight in the same population (*Phvul.006G069300*, *Phvul.008G013300*, and *Phvul.010G017600*). Other GWAS using multi-environment trials for 4 decades confirmed the involvement of genes located on chromosomes Pv02 and Pv10, and found two additional genes for seed weight, *Phvul.002G150600* and *Phvul.003G039900* (MacQueen et al. 2020). Additionally, 13 candidate genes for seed shape and size were proposed by Giordani et al. (2022) based on a GWAS conducted on a Brazilian panel of 180 accessions. However, analysis of gene expression in the Negro Jamapa genotype during seed development showed that 10,453 genes modified their expression levels, with the majority (9,701) showing decreased expression (O’Rourke et al. 2014). Many of these genes are transcription factors, although the genes involved in starch biosynthesis (e.g., *Phvul.001G082500.1*) and sucrose synthesis are highly expressed in developing seeds. Also, the high expression of abscisic acid biosynthesis genes (e.g., *Phvul.002G018700.1* and *Phvul.005G031500.1*) was observed in developing seeds, with expression decreasing as the seeds matured (O’Rourke et al. 2014). In other species, multiple pathways, including G-protein signaling, ubiquitin–proteasome pathway, mitogen-activated protein kinase (MAPK) pathway, BR signaling, transcriptional regulatory factors, and auxin signaling, are involved in the regulation of seed development (Li et al. 2019). In the ubiquitin–proteasome pathway, the ubiquitin receptor DA1 and E3 ubiquitin ligases EOD1/BB and DA2 physically interact to control seed size in Arabidopsis by regulating cell proliferation in integuments (Xia et al. 2013; Li et al. 2019). MAPK pathway consists of the different combinations of MKKK, MKK, and MAPK proteins which the plants use to regulate distinct biological processes, like plant growth, development, and defense response (Xu et al. 2018; Jiang et al. 2022), as well as could be important to regulate the grain size in rice (Xu et al. 2018; Tian et al. 2021; Wu et al. 2022). In addition, a LECTIN RECEPTOR KINASE, LecRK-VIII.2, has been reported to coordinate silique number, seed size, and seed number to determine seed yield in Arabidopsis by acting upstream of the MAPK gene (Xiao et al. 2021). All evidence indicates that the bean seed phenotype is regulated by a complex network of genes; however, there is no evidence that any loci have a higher effect on the expression of this trait.

The bean genome is available (e.g., Schmutz et al. 2014). Therefore, it is necessary to strengthen the connection between phenotype, genotype, and genome to identify annotated genes related to the expression of particular characters. The main goal of this study was to identify the consistent genomic regions and candidate genes involved in the genetic control of common bean seed size, shape, and quality traits, by combining association and differential expression studies. These analyses contribute to the understanding of the complex network of genes involved in seed phenotype control.

## Materials and methods

### Plant material

The Spanish Diversity Panel (SDP), with wide variation in seed phenotypes, was used in this study (Campa et al. 2018; https://zenodo.org/records/10263706). The SDP has homozygous lines derived from 220 landraces, most of which are from the updated Spanish Core Collection. SDP included 51 snap bean cultivars, 37 lines derived from traditional old cultivars, and well-known breeding lines. In all, 298 homozygous lines of SDP were used in this study.

**Fig. 1:**
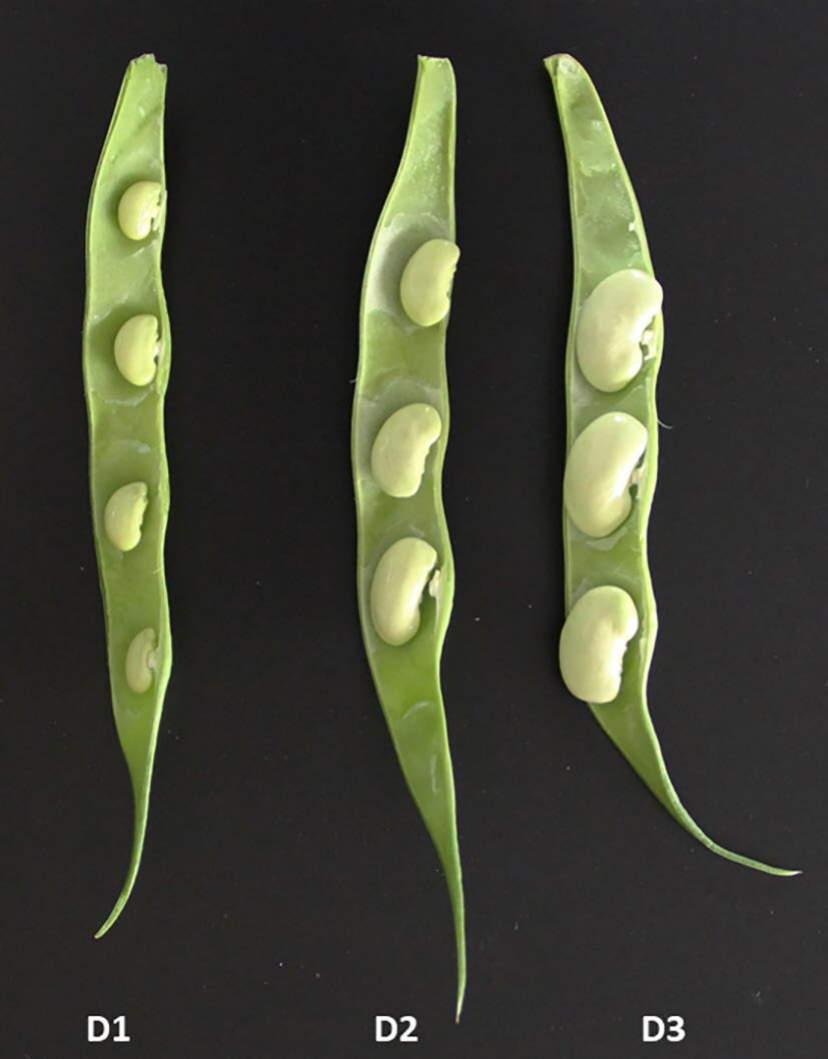
Pods of cv. Xana showing the three different growth stages of seeds used in the differential expression analysis

The genotype ‘Xana’ was used for the analysis of differentially expressed genes (DEG) during seed development (Fig. 1). The cultivar ‘Xana’ was grouped in the Andean gene pool (Campa et al. 2018). This genotype has very large white seeds, determinate growth habits, and is classified as Fabada market class. ‘Xana’ is included in the SDP as line SDP308.

### Genotyping

The genotyping-by-sequencing (GBS) method, as described by Elshire et al. (2011), was performed at BGI-Tech (Copenhagen, Denmark) using *ApeKI* restriction enzyme (Campa et al. 2018; Table S1). Sequencing reads from different genotypes were aligned using the bean genome G19833 v1.0, https://www.ncbi.nlm.nih.gov/genome/380). Genotypic data were filtered using software Tassel v5 (Bradbury et al. 2007). Lines with more than 50% missing data were removed, and in the remaining genotypes, SNP were filtered using the following criteria: (i) proportion of missing data < 10% and (ii) minor allele frequency (MAF) > 0.05. SNPs were named according to their physical position in the bean genome G19833 v1 (chromosome followed by the physical position in base pairs).

### Phenotyping

SDP was grown in a greenhouse in Villaviciosa, Spain (43°29′01′ N, 5°26′11′ W; elevation 6.5 m) for three seasons (2016, 2017, and 2018). The experimental design was a randomized complete design in which there was a replicate with a single 1 m row plot, including 8–10 plants per line. Standard agronomic practices for tillage, irrigation, fertilization, and pest control were followed to ensure adequate plant growth and development. Phenotyping was conducted for seven seed traits: seed shape (area, length, width, and length-to-width ratio), seed weight, and seed quality after rehydration (coat proportion and water absorption) (Table 1). Seed dimensions were digitally recorded and analyzed using SmartGrain software (Tanabata et al. 2012). The trait 25-seed weight was manually recorded. Coat proportions and water absorption traits were recorded manually according to Castellanos et al. (1995).

**Table 1:** Description of the seven analyzed seed traits. Codes for each character are indicated in parentheses

Trait	Unit	Method*	Description
Seed area (SA)	mm^2^	2	Measure of 25 randomly chosen seeds
Seed length (SL)	mm	2	Measure in parallel to the hilum of 25 randomly chosen seeds
Seed width (SWI)	mm	2	Measure perpendicular to the length of 25 randomly chosen seeds
Seed SL/SWI ratio (LWR)		2	Ratio SL/SWI
25-seed weight (SW)	g	1	Measure of four sets of 25 dry seeds
Water absorption (WA)	%	1	Average of a set of 25 seeds per plot without considering non-rehydrated seeds (according to (Castellanos et al. 1995))
Coat proportion (CP)	%	1	Average of a set of 10 seeds per plot (according to (Castellanos et al. 1995)

*(1) manually measured; (2) measured from digital images with the help of the software SmartGrain

### Statistical analysis seed traits

All statistical analyses were performed using R software version 4.3.0 (R Core Team 2023). Outliers were removed before mean estimation using the mode ‘blup’ in the phenotype package (Piepho et al. 2008). Phenotypic variation in individual traits was visualized using the probability density distribution generated by the ggplot2 package (Wickham 2016). Spearman’s correlation coefficients between the seven traits were calculated using the R corrplot package (Wei and Simko 2021).

Hierarchical clustering on principal components (HCPC) analysis was performed to identify the main clusters in which bean lines could be grouped based on the genotypic data. This analysis was performed using R software with the packages FactoMineR and FactoExtra (Lê et al. 2008). Putative significant differences in seed traits among the clusters established from the HCPC analysis were investigated using ANOVA, followed by a post hoc Tukey test.

### GWAS and haplotype block detection

Association analysis was performed using the FASTmrEMMA model (Wen et al. 2018), implemented in the mrMLM package (Zhang et al. 2020) of the R project (R Core Team 2023). Principal component analysis (PCA) and the kinship matrix obtained by the centered-IBS method were considered to account for multiple levels of relatedness within the lines included in the panel. A restricted maximum likelihood (REML) and critical LOD score of 3 were considered critical thresholds of significance for the identification of significant associations trait-SNP (QTN).

Haplotype blocks around QTNs were investigated using the Haploview 4.2 software (Barrett et al. 2005) with default software parameters and algorithms. The identified blocks were named using the prefix ‘Seed,’ chromosome number, and start position in Mb. To complete the characterization of the delimited blocks revealed by GWAS, the genomic positions in the bean genome were compared with those of QTL previously reported in common bean (González et al. 2016; Murube et al. 2020; Berry et al. 2020; Bassett et al. 2021; Giordani et al. 2022; Ugwuanyi et al. 2022; Arriagada et al. 2022). The list of annotated genes under the QTL was established based on those residing within the region delimited by the leftmost and rightmost flanking SNP in the defined haplotype blocks.

### RNA-seq

Plants of the genotype ‘Xana’ were grown in pots of 7 litters during the summer of 2022 under greenhouse conditions. Seeds collected at three different growth stages were used to identify differentially expressed genes by RNA-seq (see Fig. 1): D1, the beginning of seed development (seeds with 0.8–1 cm length and green color); D2, intermediate development stage (1.5–2 cm length and green color); and D3, final development stage (2–2.5 cm length and green–white appearance). The experimental design included two biological replicates corresponding to two seed samples from different plants and pods.

The seeds were extracted from the pods, flash-frozen in liquid nitrogen, and stored at − 80 °C before RNA extraction. Total RNA from two biological replicates per growth stage was isolated using the RNeasy Plant Mini Kit, following the manufacturer’s instructions (Qiagen, Germany). RNA was quantified using fluorometric methods, and the quantity was determined using a 2100 Bioanalyzer Instrument (Agilent Technologies, UK). RNA libraries were prepared using the TruSeq Stranded mRNA Sample Preparation Kit (Illumina), and sequencing was performed on the Illumina platform (Macrogen, Korea).

The reads were mapped to the reference genome G19833 v1.0 (Schmutz et al. 2014) using HISAT2 splice-aware aligner (Kim et al. 2015). Expression profiles were represented as read counts and normalized by calculating the Trimmed Mean of M-values (TMM). Genes with expression levels less than 33% were removed. A principal component analysis (PCA) was performed to detect the possible sources of noise in the results. The NOISeqBIO function of the NOISeq package in R (Tarazona et al. 2015) was used to identify differentially expressed genes (DEG) through comparisons at different growth stages: D3 versus D1, D2 versus D1, and D3 versus D2 (see Fig. 1). DEGs were identified using *q* > 0.99. Specific and common DEGs among the three comparisons were detected and visualized using Venn diagrams constructed using the package ggVenn/ggplot2 (Wickham 2016).

### Gene ontology enrichment analysis

Gene ontology (GO) annotation was done using the ‘Phaseolus vulgaris’ organism database in AnnotationHub resource (Morgan and Shepherd 2023) considering the three categories: biological process (BP), molecular function (MF), and cellular components (CC) to investigate the functional groups of the observed DEGs. Overrepresentation analysis (ORA) of candidate genes was performed using the R package clusterProfiler (Wu et al. 2021) based on the hypergeometric test (*p* value) and Benjamin–Hochberg method for controlling the false discovery rate (*q* value).

### Approach to candidate genes

The identification of potential candidate genes was focused on considering the following criteria: (i) The QTL regions revealed by this study and colocalized with previously reported QTL for the same traits; (ii) DEGs located in the genomic regions delimited in this study; (iii) match with the candidate genes for domestication events previously proposed by Schmutz et al. (2014); (iv) match with the reported DEGs during seed development from the common bean expression atlas (O’Rourke et al. 2014).

Complete genomic sequences of the selected putative candidate genes were obtained from the Phytozome v12 database (Mesoamerican genomes UI111 v1.1, Labor Ovalle1.1 and 5 593v1.1). The sequences obtained were analyzed by alignment (BLASTn) with sequences of the reference genome G19833 v1.1, using default parameters. The polymorphisms identified were nucleotide variation, insertions, deletions, and the number of predicted genes.

## Results

### Genotyping of the SDP

Genotyping of 298 lines included in the Spanish Diversity Panel (SDP) was filtered considering homozygous sites, missing values (< 10%), and minor allele frequency (> 0.05), resulting in 4,658 SNPs distributed across the 11 bean chromosomes (Table S1; Figure S1). The number of SNPs per chromosome ranged between 298 (Pv10) and 619 (Pv02) SNPs.

### Phenotypic variation

**Fig. 2:**
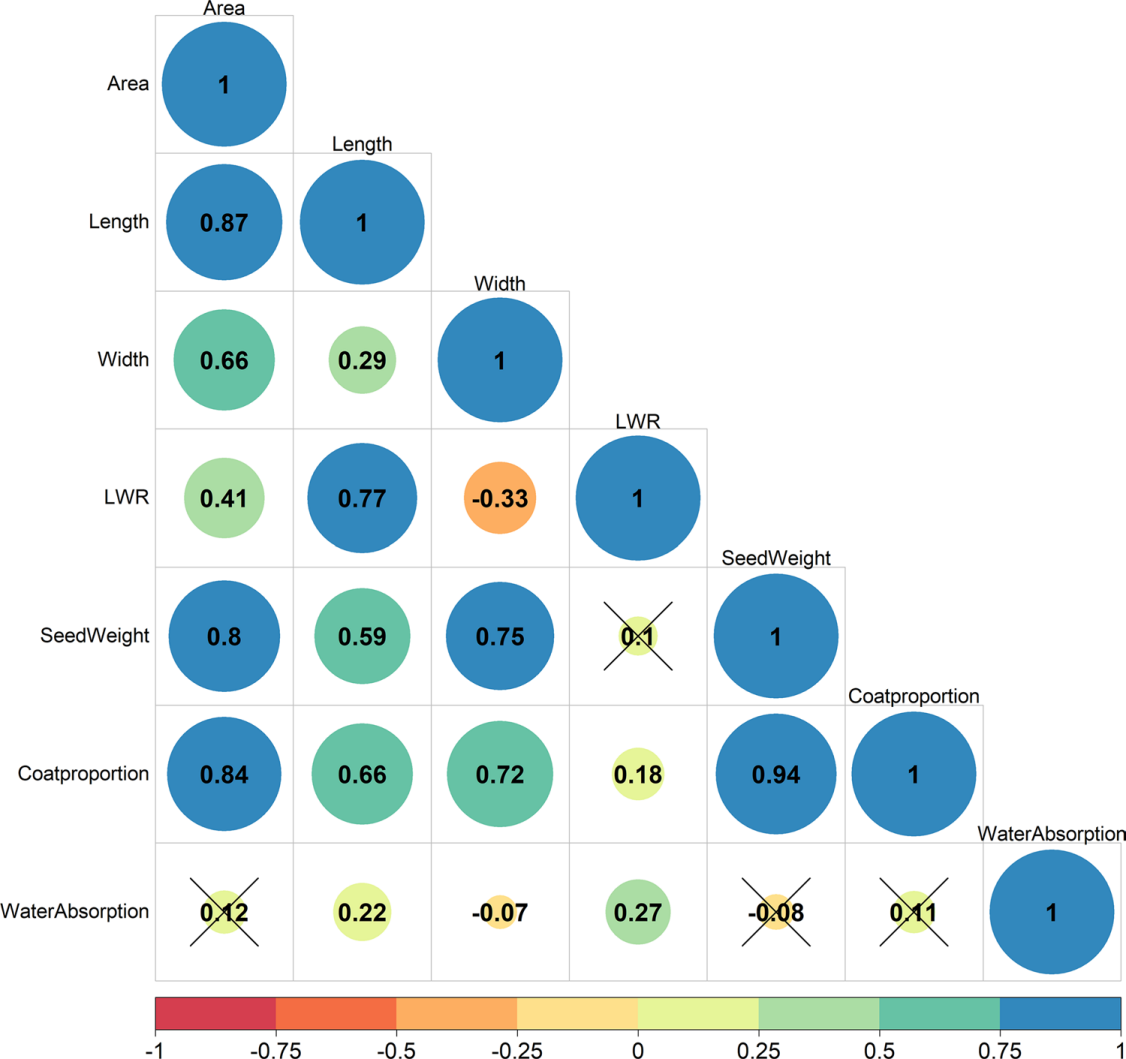
Spearman’s correlation coefficient between the evaluated seed traits. Black crosses represent not significantly associated (*p* > 0.05)

Table 2 shows the observed variation in the seven traits evaluated. Phenotypic evaluation of the SDP panel revealed a wide variation in all cases (see Figure S2). For instance, SL and SW ranged from 8.68 mm (observed in SDP262) to 22.41 mm (SDP308), and from 3.9 g (SDP009) to 27.59 g (SDP308), respectively (see Table S2). Similarly, WA showed wide variation in this panel, ranging from 39.3% (SDP083) to 63.3% (SDP106). All traits showed a good fit to a normal distribution (Kolmogorov–Smirnov test), except for LWR (Figure S2). Significant correlations were detected in most cases, except for SW, LWR, WA, SA, SW, and CP (Fig. 2). Correlations (*r*) ranged from − 0.07 to 0.94 and were positive in most cases. Overall, 11 of the 21 trait combinations showed moderately positive correlations (*r* > 0.4).

**Table 2:** Observed variation in the seven seed traits analyzed in the Spanish Diversity Panel (SDP). Means, standard deviations (SD), and variation intervals (min–max) are indicated

Seed trait	Mean	SD	Min–max
Area (mm^2^)	86.86	23.72	41.46–184.37
Length (mm)	13.61	2.41	8.68–22.41
Width (mm)	7.97	1.03	5.00–10.59
SL/SWI ratio	1.73	0.34	1.21–2.98
25-seed weight (g)	12.53	4.14	3.90–27.59
Coat proportion (%)	10.41	3.36	3.37–25.38
Water absorption (%)	51.49	3.11	39.26–63.35

### Genome-wide association study

Association analysis revealed 38 SNP-trait-significant associations (QTN), although 11 SNPs were associated with more than one character. The 27 unique SNPs were located on 10 chromosomes (all except for chromosome Pv06). The distribution and characteristics of QTN are presented in Table 3 (see Figure S3). For instance, the six QTN for SW were located on chromosomes Pv01, Pv03, Pv04, Pv07, Pv08, and Pv10, whereas those for SL were located on chromosomes Pv04, Pv07, Pv08 (two regions), Pv10, and Pv11.

**Table 3:** Characteristics of the significant associations (LOD > 3) SNP-traits identified using the FASTmrEMMA method in the SDP for seven seed traits

SNP	Seed trait	Chr	QTN effect	LOD score	− log_10_(*p*)	*r*^2^ (%)	MAF
s1_50842559	CP	Pv01	1.78	6.09	6.92	4.46	0.21
s1_52137885	SW	Pv01	− 1.4	3.69	4.42	1.63	0.18
s2_28212493	LWR	Pv02	− 0.1	3.64	4.37	2.14	0.45
s2_37004268	SWI	Pv02	0.38	3.71	4.44	3.01	0.36
s2_39810114	LWR	Pv02	− 0.19	11.3	12.26	7.13	0.42
s3_46955356	SA	Pv03	16.12	6.09	6.93	9.1	0.29
s3_46955356	SWI	Pv03	− 0.87	11.34	12.3	9.8	0.17
s3_47996582	SW	Pv03	− 3.3	12.17	13.15	8.74	0.17
s3_47996582	CP	Pv03	− 2.54	10.37	11.32	7.88	0.17
s4_26787677	SA	Pv04	− 14.89	11.61	12.58	7.96	0.29
s4_26787677	SL	Pv04	− 1.03	4.96	5.76	3.66	0.29
s4_26787677	SW	Pv04	− 0.95	3.06	3.76	1.07	0.29
s4_43792143	LWR	Pv04	0.15	5.49	6.31	3.79	0.27
s5_3286816	WA	Pv05	2.11	4.92	5.72	10.82	0.41
s5_37260589	WA	Pv05	1.37	4.12	4.88	4.17	0.33
s5_37715374	LWR	Pv05	− 0.26	9.88	10.82	3.02	0.06
s7_633265	SL	Pv07	0.88	5.63	6.45	3.11	0.39
s7_663226	SW	Pv07	2.09	7.23	8.1	5.02	0.29
s7_3895030	LWR	Pv07	0.08	3.4	4.12	1.48	0.44
s7_557444	CP	Pv07	1.79	4.54	5.32	6	0.32
s8_55946412	SA	Pv08	11.05	6.02	6.85	3.46	0.2
s8_55946412	SL	Pv08	1.21	6.16	6.99	4	0.2
s8_55946412	LWR	Pv08	0.11	5.22	6.03	1.78	0.2
s8_55946412	SW	Pv08	2.08	7.05	7.91	4.02	0.2
s8_55946412	CP	Pv08	1.59	4.99	5.79	3.56	0.2
s9_12670503	SWI	Pv09	0.39	3.1	3.8	1.86	0.16
s9_31015943	LWR	Pv09	0.19	8.04	8.93	2.14	0.08
s10_32685348	WA	Pv10	1.43	5.08	5.87	5.01	0.44
s10_39185557	SA	Pv10	− 24.84	5.94	6.77	9.1	0.09
s10_39185557	SL	Pv10	− 1.77	4.72	5.5	4.43	0.09
s10_39193928	SW	Pv10	− 3.5	5.34	6.15	5.91	0.1
s10_39193928	CP	Pv10	− 2.7	4.68	5.46	5.35	0.1
s10_40537163	SA	Pv10	− 16.53	6.4	7.25	6.32	0.16
s10_40537163	SL	Pv10	− 1.55	5.2	6	5.32	0.16
s10_40979207	SWI	Pv10	− 0.63	5.6	6.42	4.8	0.15
s11_1587588	SL	Pv11	− 1.06	6.57	7.42	4.57	0.4
s11_1701280	SWI	Pv11	− 0.47	5.96	6.79	3.23	0.22
s11_2105912	SA	Pv11	− 12.36	7.05	7.92	5.02	0.25

**Fig. 3:**
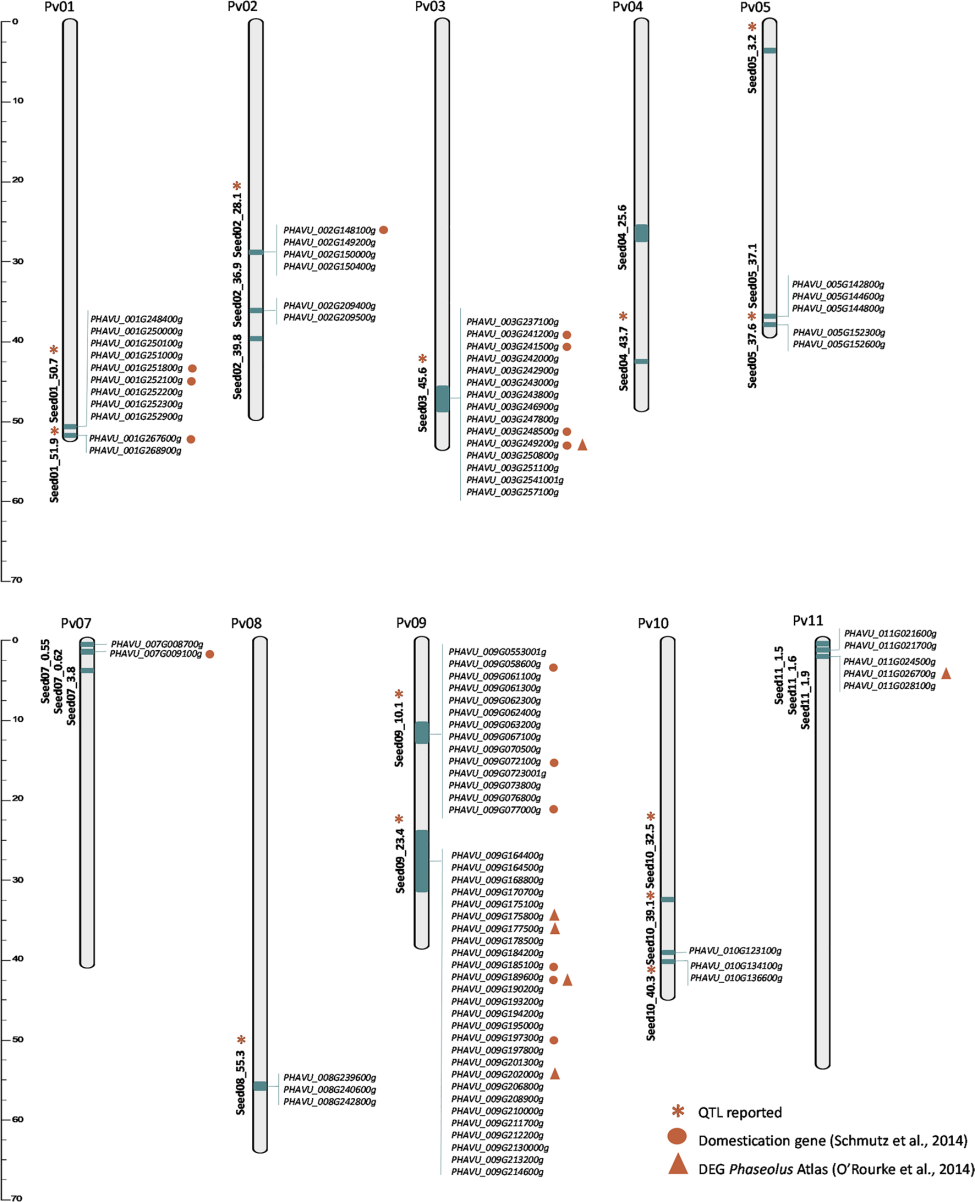
Chromosomal positions of genomic regions associated with seven seed traits identified by GWAS (green boxes). * Regions colocated with previously reported QTL for seed traits. Genes differentially expressed during seed development underlying those genomic regions revealed by RNA-seq analysis are shown at the right of each chromosome

Linkage disequilibrium (LD) analysis showed that the 27 SNP were organized in 23 genomic regions (blocks) ranging between 5 bp (Seed02_39.8) and 8.4 Mbp (Seed09_23.4) (Table 4). The QTL region Seed11_1.5 was tagged with an SNP. The positions of the genomic regions were compared to previously reported QTL regions associated with seed traits. Seven studies on biparental populations and diversity panels were considered, and 34 QTL associated with the genetic control of seed traits were found, revealing 13 overlapping regions on chromosomes Pv01, Pv02, Pv03, Pv04, Pv05, Pv08, Pv09, and Pv10 (Table 4). Interestingly, eight regions (most of which were associated with SW) were detected in more than one study: Seed01_50.7, Seed01_51.9, Seed02_28.1, Seed03_45.6, Seed08_55.3, Seed09_10.1, Seed09_23.4, Seed10_39.1, and Seed10_40.3 (see Fig. 3).

**Table 4:** The genomic regions carrying the identified SNPs were revealed using linkage disequilibrium analysis. Correspondence with reported QTL for similar traits is shown. The reference of the reported QTL (Ref.), the numbers of reported DEG (Rep. DEG), and observed DEG (Ide. DEG) in each genomic region are also indicated

QTL name	Seed trait	Genomic region		QTL reported			Gene expression		
		Start	End	Traits	QTL name	Ref.*	Num. genes	Ide. DEG	Rep. DEG
Seed01_50.7	CP	50,711,659	51,126,515	CP; SW; SCP	SPE1.1; SW-1^AM^; qProtein-a	1; 4; 7	73	9	2
Seed01_51.9	SW	51,943,288	52,159,049	SW; SPC	SW1.3; qProtein-a	4; 7	25	2	1
Seed02_28.1	LWR	28,125,848	29,131,665	SW; CP	SW-1^MA^; Yd_MQTL2.4; SW.2.2; SCP.2.2	1; 2; 5	62	4	4
Seed02_36.9	SWI	36,917,430	37,025,044				12	2	1
Seed02_39.8	LWR	39,810,109	39,810,114				0	0	
Seed03_45.6	SA	45,689,287	48,502,614	SW	SW3.3; SW3.1	2; 4	234	15	16
	SWI								
	SW								
	CP								
Seed04_25.6	SA	25,641,883	27,416,740				24	0	1
	SL								
	SW								
Seed04-43.7	LWR	43,792,143	43,865,501	SW	SW4.1^SA^	1	8	0	0
Seed05_3.2	WA	3,286,515	3,324,361	WA	WU.5.1	2	6	0	0
Seed05_37.1	WA	37,141,875	37,376,050				26	3	0
Seed05_37.6	LWR	37,685,717	37,988,349	SW	SW-5^MA^	1	35	2	3
Seed07_0.55	CP	557,444	620,707				9	1	1
Seed07_0.62	SL	627,376	1,090,742				76	1	5
	SW								
Seed07_3.8	LWR	3,895,030	3,895,149				1	0	0
Seed08_55.3	SA	55,393,031	56,063,905	SW; SWI; SA	SW8.3; SW8.1^AN,SA^; qSDia-b; S1_379992973	1; 4; 6; 7	66	3	4
	SL								
	LWR								
	SW								
	CP								
Seed09_10.1	SWI	10,190,403	12,983,869	SW; CP; SP; SL	Yd_MQTL9.1; SCP.9.1; SP9^XB^; SL9^XB^	3; 2; 5	263	14	23
Seed09_23.4	LWR	23,402,412	31,822,063	SW; SWI	qSDia-c; Yd_MQTL9.2	7; 5	559	27	43
Seed10_32.5	WA	32,549,846	32,685,348	WA; SW	WU10.1; SW10.1	4	4	0	1
Seed10_39.1	SA	39,180,865	39,358,330	SW; SWI; SL	SW10.1; qSDia-e; Yd_MQTL10.2; SL10^XC^	3; 4; 7; 5	18	1	2
	SL								
	SW								
	CP								
Seed10_40.3	SA	40,365,242	40,979,207	SWI	SWI10^XC^	3	45	2	2
	SL								
	SWI								
Seed11_1.5	SL	1,587,588	1,587,588				1	0	
Seed11_1.6	SWI	1,657,560	1,701,280				8	2	1
Seed11_1.9	SA	1,949,116	2,323,534				50	3	8

*(1) González et al. (2016), (2) Berry et al. (2020), (3) Murube et al. (2020), (4) Bassett et al. (2021), (5) Arriagada et al. (2022), (6) Giordani et al. (2022), (7) Ugwuanyi et al. (2022)

### Seed weight QTL dissection

**Fig. 4:**
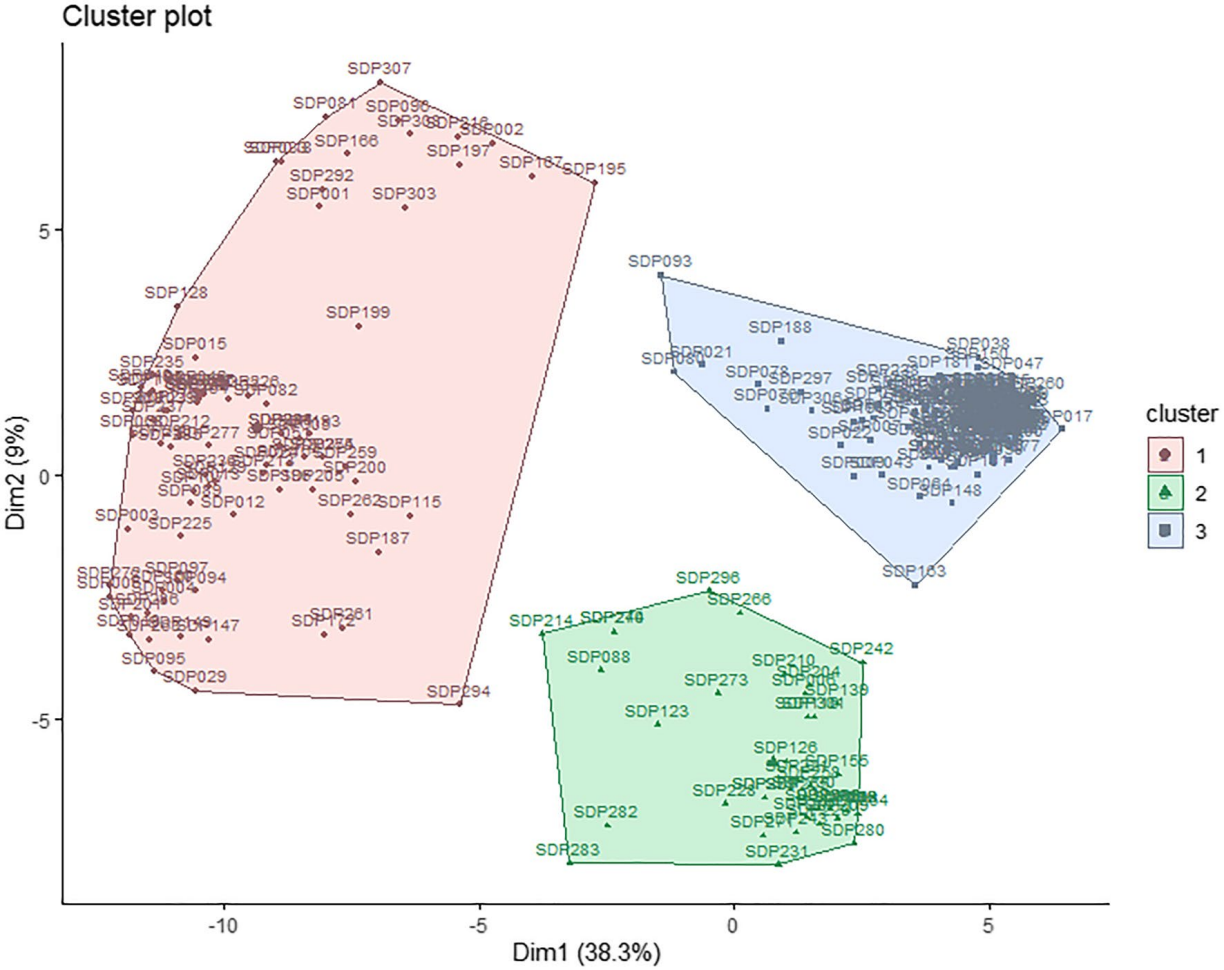
Biplots showing the results of hierarchical clustering on principal components using 102 SNP tagging the five consistent QTL regions associated with SW

Five QTL associated with SW have been consistently identified in other studies: Seed01_51.9, Seed03_45.6, Seed07_0.62, Seed08_55.3, and Seed10_39.1. A total of 102 SNPs that tagged these five QTL were selected, and hierarchical clustering on principal components (HCPC) analysis was performed with these SNPs revealing two main dimensions that explained 47.3% of the variance and led to the establishment of three main clusters with the SDP lines (Fig. 4; Table S2).

Cluster 1 is formed by 85 lines, including typical Mesoamerican genotypes such as Sanilac (SDP290), Cornell49242 (SDP225), IVT7214 (SDP248), and AB136 (SDP005). The group had an average weight of 10.54 g per 25 seeds and contained 16 lines classified in the intermediate population and 1 classified in the Andean population (Campa et al. 2018).

Cluster 2 is formed by 43 lines, 38 of them classified as intermediate between both gene pool. The group had an average weight of 10.37 g per 25 seeds and did not differ significantly from cluster 1. Many of these lines are snap bean cultivars such as Fin de Bagnols (SDP232), Triomphe de Farcy (SDP293), Gloire De Saumur (SDP242), and Manteca de los Mercados (SDP247).

Cluster 3 consisted of 170 lines, of which 141 were classified in the Andean gene pool. This group included the typical Andean cultivars Tendergreen (SDP295), MDRK (SDP256), Perry Marrow (SDP276), and G19833 (SDP238), one of the bean genomes available (Schmutz et al. 2014). Cluster 3 included 28 lines classified as intermediate between both gene pools, and a line grouped in the Mesoamerican gene pool (SDP080). The mean seed weight was 14.06 g in this group, which differed significantly from that of clusters 1 and 2.

An analysis was also carried out for each QTL region to evaluate the effect of each region on seed weight. HCPC analysis showed three groups for each QTL region (Figure S4). The percentage of explained variance varied between 52.3% (Seed07_0.62) and 89% (Seed01_51.9). Significant differences in SW were detected between the groups of lines established for each QTL (Table 5). The QTL regions Seed01_51.9 and Seed10_39.1 showed the greatest differences between the two groups with extreme mean values (6.63 and 6.47 g, respectively) and significant differences in the means of the three groups.

**Table 5:** Mean values for 25-seed weight in the three clusters obtained from the HCPC analysis using SNP tagging of the 5 consistent QTL regions associated with seed weight (see Figure S4). Results of analysis of variance (ANOVA)

	5 QTL	Seed01_51.9	Seed03_45.6	Seed07_0.62	Seed08_55.3	Seed10_39.1
Num. SNP	102	7	42	20	21	12
Percentage explained variance (%)	47.30	89	67.30	52.30	73.40	68.90
*Cluster 1*						
Mean SW	10.54^a^	13.02^a^	13.26^a^	13.34^a^	9.27^a^	9.24^a^
Num. lines	85	118	211	215	45	89
*Cluster 2*						
Mean SW	10.37^a^	16.80^b^	8.22^b^	14.09^a^	10.34^a^	15.71^b^
Num. lines	43	55	39	9	68	29
*Cluster 3*						
Mean SW	14.06^b^	10.17^c^	12.78^a^	9.94^b^	14.11^b^	13.63^c^
Num. lines	170	125	48	74	185	180
ANOVA	9.8e^−14^***	< 2e^−16^***	< 2.92e^−12^***	1.4e^−9^***	< 2e^−16^***	< 2e^−16^***

^a^, ^b^, and ^c^ are homogeneus groups defined by the post-hoc Tukey tests. *, *p* < 0.05; **, *p* < 0.01; ***, *p* < 0.001

### Differentially expressed genes

**Fig. 5:**
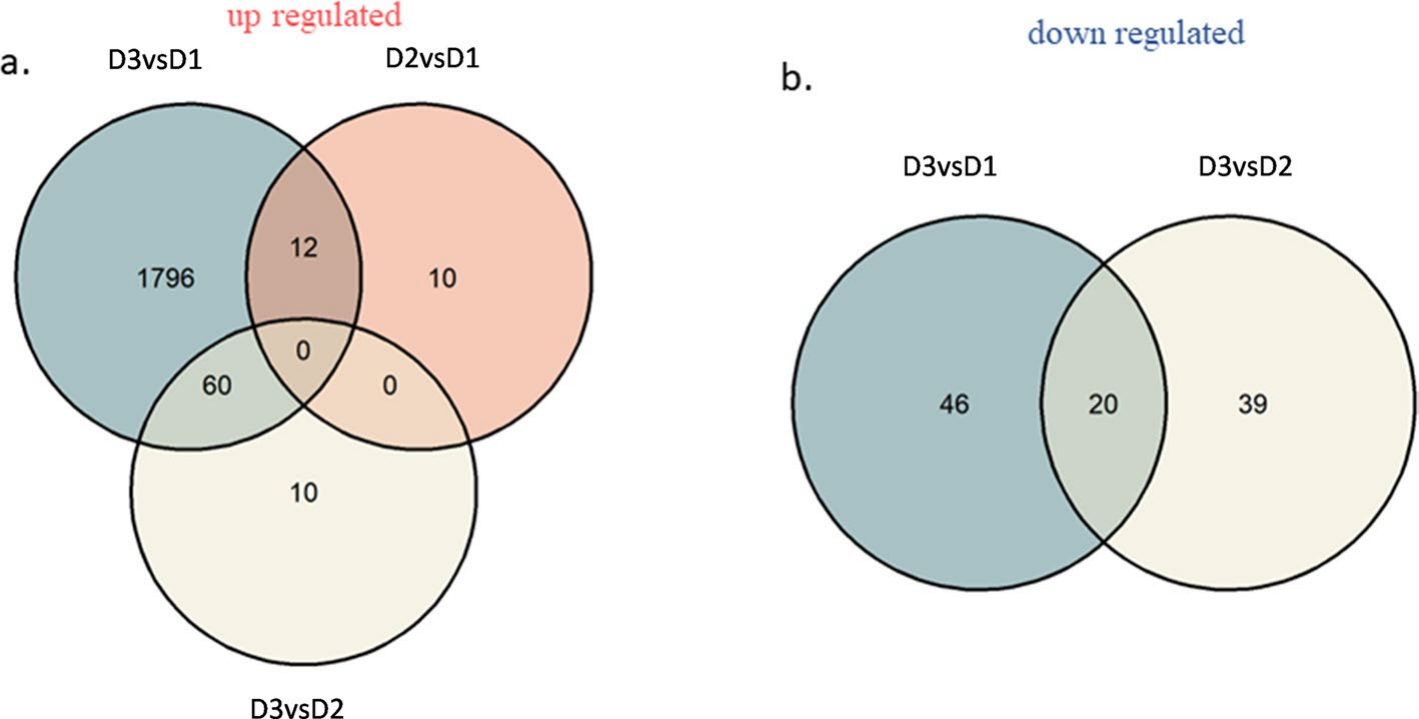
Venn diagrams showing DEGs detected in each comparison (D2 vs. D1, D3 vs. D1, D3 vs. D2). **a** Up-regulated genes. **b** Down-regulated genes

The read counts for the expression levels at each seed development stage (D1, D2, and D3) and the replicates per locus are shown in Table S3. The reads from all samples were used for transcriptome assembly, and an average of 91.2% of the reads were mapped to the reference genome. The mapped reads were normalized by calculating the TMM-normalized reads, which revealed two components that explained 67% of the variation (Figure S5a). The obtained plot shows the grouped samples, except in the case of a sample derived from the D1 development stage (Figure S5b). Differentially expressed genes (DEGs) were identified by comparing three stages of seed development. (D2-D1; D3-D1; D3-D2; see Fig. 1). In total, 2,085 differentially expressed genes involving 1992 unique genes were identified in this analysis (Table S4). The majority of DEGs were up-regulated (1,888) (Fig. 5), and most of them were detected when the comparison was made between stages D3 and D1. Down-regulated DEGs were not detected in the comparison between the development stages, and up-regulated DEGs common among the three stages were also not detected.

### GO enrichment analysis of DEGs

Gene ontology (GO) enrichment analysis was performed with 1,934 genes, corresponding to the DEGs in D3 compared to D1. The biological process (BP) and cellular component (CC) categories showed enrichment terms, whereas the molecular function (MF) category did not (Table S5). For BP, 19 GO terms were enriched, with more important terms related to intracellular cell establishment and functions, such as protein transport, protein and macromolecule localization, and compound metabolic processes (Figure S6a). For CC, 34 terms were enriched, most of which were implicated in functions related to endoplasmic reticulum or coated COPI-coated vesicles (Figure S6b).

### Putative candidate genes for seed traits

The 23 QTL contained 1,605 annotated genes in the G19833 genome, and 91 genes were differentially expressed in RNA-seq analysis during seed development (Table S6). These 91 differentially expressed genes were located in only 16 QTL regions (Table S7). The number of DEGs per region ranged between one (Seed10_39.1) and 27 (Seed09_23.4). Six of these 91 genes were described in the gene expression atlas during seed development: *PHAVU_003G249200g, PHAVU_009G175800g, PHAVU_009G177500g, PHAVU_009G189600g, PHAVU_009G202000g,* and *PHAVU_011G026700g* (O’Rourke et al. 2014; Table S6). In addition, 15 of these 91 genes have been reported as relevant genes in the domestication process by Schmutz et al. (2014). Functional annotation of these 91 DEG revealed molecular functions and biological processes already reported in seed development (see Table S7).

The reference bean genome (G19833) contained 22 DEGs located in five consistent QTL regions associated with SW. The genomes of Labor Ovalle, UI111, and 5-593 had predicted homologous genes for each of these DEGs, except for *PHAVU_010G123100g*, which had two genes (Table S8). The respective sequences of these genes were aligned, and different types of variation were observed, compared to the reference genome: mismatches, insertions, deletions, and duplications. The genome of 5-593 presented less variation than that of Labor Ovalle and UI111 compared to the genome of G19833. However, the levels of variation were not the same for the 22 DEGs. Thirteen of the 15 predicted genes located on chromosome 3 showed a very low variation with G19833. In contracts, eight of the 22 genes showed high variation in the three genomes in the respective alignments with G19883: *PHAVU_001G267600g, PHAVU_001G268900g, PHAVU_003G241500g, PHAVU_007G009100g, PHAVU_008G239600g, PHAVU_008G240600g, PHAVU_008G242800g*, and *PHAVU_010G123100g*. For example, the three homologous genes of *PHAVU_008G240600g* had an insertion of 46 bp.

## Discussion

Seed phenotype is a relevant characteristic of both dry and snap beans. While many studies have focused on seed coat color inheritance, there is limited research on the genetic control of seed size, seed shape, and seed quality traits, as well as limited data on the candidate genes involved. In this study, association and differential expression analyses were combined to identify consistent genomic regions and candidate genes controlling those seed traits. For this purpose, the seed variation for seven characters was evaluated in a diverse panel, the Spanish Diversity Panel (Campa et al. 2018). The results confirmed a continuous distribution of the seven traits, which is in agreement with the results of previous studies. In addition, most evaluated characters were positively and significantly correlated, suggesting the involvement of common loci in genetic control. The exception was WA, which did not show significant correlations with SA, SW, and CP (see Fig. 2). Seed weight, a yield-related trait, was positively correlated with shape traits (SA, SL, and SWI) and the seed quality trait CP.

GWAS and LD analysis identified 23 regions that were associated with seven seed traits. Eighteen regions were associated with seed size and shape traits on ten chromosomes while five regions were only associated with quality traits (two for CP and three for WA). Well-characterized QTL, consistent among backgrounds, well delimited and tagged, and with a significant contribution to the expression of the trait are desirable for use in breeding. A few studies have reported QTL for seed size and shape (González et al. 2016; Murube et al. 2020; Bassett et al. 2021; Giordani et al. 2022; Ugwuanyi et al. 2022; Arriagada et al. 2022) and seed quality traits (Berry et al. 2020; Bassett et al. 2021). The results allowed the identification of 13 consistent genomic regions associated with QTL that were previously reported in different genetic backgrounds and environments, indicating the relevance of such regions in the control of seed characters. The SNP s8_55946412 tagging the QTL region Seed08_55.3 was also associated with seed area (Giordani et al. 2022). Two of these QTL regions (Seed08_55.3 and Seed10_39.1) were associated with more than three traits, which agreed with the observed correlations. The QTL regions Seed05_3.2 and Seed10_32.5 were associated with water absorption on chromosomes Pv05 and Pv10. A QTL for WA was previously described by Berry et al. (2020) at the position of Pv10. QTL Seed09_23.4, tagged by 86 SNPs and associated LWR, was the largest QTL region identified. The size of QTL can depend on factors such as variation among genomes, density of markers, recombination of the region, or number of genes that contribute to the characteristics. The QTL region Seed09_23.4 was also involved in SW and SWI (Ugwuanyi et al. 2022; Arriagada et al. 2022). Interestingly, this delimited region annotated the genes *Phvul.009G190100* and *Phvul.009G202100*, which had a significant effect on yield in a set of 42 common bean genotypes evaluated at two locations (Reinprecht et al. 2021). Moreover, seven of the 115 genes previously related to seed weight and domestication by Schmutz et al. (2014) were located in seven regions associated with seed size and shape traits.

Seed size plays a crucial role in the domestication of the common beans. The wild type has smaller seeds than domesticated ones, and domesticated Mesoamerican populations are smaller in size (Singh et al. 1991; Chacón‑Sánchez 2018). Choosing the 102 SNP underlaying the five consistent QTL regions for SW (Seed01_51.9, Seed03_45.6, Seed07_0.62, Seed08_55.3, and Seed10_39.1), three groups could be established from the HCPC analysis, two of which corresponded well with the Andean and Mesoamerican gene pools, and a third with intermediate material between both gene pools (Campa et al. 2018). The results indicated that the lines included in the Andean group had significantly heavier seeds than those included in the Mesoamerican group. In addition, the effects of each of the five QTL on SW were investigated. QTL dissection showed a significant effect on seed weight in the five consistent QTL regions. HCPC analysis based on the SNP genotype of each QTL region revealed three main groups, which significantly differed in SW. Remarkably, differences were established by the QTL regions Seed01_51.9 and Seed10_39.1, 6.63 g (Clusters 2 and 3) and 6.47 g (Clusters 1 and 2) between the two extreme groups (see Table 5). Both regions were found to be associated with seed weight in several QTL analyses (Bassett et al. 2021; Ugwuanyi et al. 2022; Arriagada et al. 2022); therefore, they are of special interest for breeding. Interestingly, the end of chromosome Pv01 is important for bean domestication and adaptation because it contains genes for determinacy (*Fin* locus) and sensitivity to photoperiod (*Ppd* locus; Weller et al. 2019).

The WA and CP of bean seeds are highly correlated with cooking time (Elia et al. 1997; Cichy et al. 2015). Seed cooking time in common beans is an important trait for consumer preference, with implications for nutrition, health, and the environment; the long cooking time of common beans is a major hindrance to its widespread consumption (Diaz et al. 2020; Haman et al. 2020). The Seed01_50.7 region, associated with CP in this study, was described as a QTL for seed content protein by Ugwuanyi et al. (2022), and the genes *PHAVU_001G251000g* and *PHAVU_001G252200g* were annotated at this position. The gene *PHAVU_001G251000g* encodes an inositol/myoinositol phosphatase synthase, a molecule precursor to a large variety of compounds and has been implicated in seed development in other legumes (Hegeman et al. 2001; Chiera and Grabau 2007). The gene *PHAVU_001G252200g* encodes an asparagine synthase, which is an amino acid positively correlated with protein concentration in soybean seeds (Pandurangan et al. 2012). The Seed10_39.1 region, which is also associated with CP, contains the gene *PHAVU_010G123100g*, which encodes a pectinesterase inhibitor involved in seed coat development in Arabidopsis. WA rate is also related to the emergence and germination of plants (Powell et al. 1986; Vidak et al. 2022), and some putative QTLs that control this trait have been reported (Cichy et al. 2015; Diaz et al. 2020; Berry et al. 2020; Bassett et al. 2021). The region Seed05_37.1, found in this work, associated with WA was reported by Berry et al. (2020); also, the region Seed10_32.5 was previously described as WA associated by Bassett et al. (2021).

Seed phenotype is the result of seed development. Two distinct phases during seed development have been described in legumes: The first phase involves cell division in the embryo, followed by a second phase, which regulates seed thickness via cell expansion and is highly influenced by the environment (Domoney et al. 2006). Changes in the transcriptomic profile of the ‘Xana’ cultivar during seed development were studied and 1,992 DEGs were detected. Many DEGs were found in the comparison between development stages D3 and D1. GO enrichment analysis revealed GO enrichment only in the categories of biological processes and cellular components. The enriched GO terms have important functions in plant development processes, such as those related to the Golgi apparatus, endoplasmic reticulum, and coated vesicles, which are essential for plant growth (Ahn et al. 2015). In all, 91 of 1,992 DEGs were located under the 23 QTL regions and 15 of them (*PHAVU_001G251800g; PHAVU_001G252100g; PHAVU_001G267600g; PHAVU_009G072100g; PHAVU_003G241200g; PHAVU_003G241500g; PHAVU_003G248500g; PHAVU_003G249200g; PHAVU_007G009100g; PHAVU_009G058600g; PHAVU_009G072100g; PHAVU_009G07700g; PHAVU_009G185100g; PHAVU_009G189600g; PHAVU_009G197300g*) were considered associated to domestication events by Schmutz et al. (2014). On the other hand, six DEG identified in this study located underlying some QTL regions were also identified as DEG in seed development by O’Rourke et al. (2014): *PHAVU_003G249200g, PHAVU_009G175800g, PHAVU_009G177500g, PHAVU_009G189600g, PHAVU_009G202000g, and PHAVU_011G026700g*. Identification of the same genes in different studies consolidates their involvement in controlling seed phenotypes. The annotated function of these 91 DEG in the QTL region agreed with functions already reported in seed development. For example, functions related to ubiquitin activities are known to determine seed size in Arabidopsis and rice (Li and Li 2014); the gene *PHAVU_002G148100g* encodes a ubiquitin hydrolase; the gene *PHAVU_003G250800g* encodes a ubiquitin receptor DA1, which in Arabidopsis thaliana controls seed and organ growth by restricting cell proliferation (Li and Li 2014); and the gene *PHAVU_005G142800g* encodes a ubiquitin ligase similar to the E3 ligase EOD1/BB identified as a negative regulator of seed size (Li et al. 2008). Across these 91 genes, we also found functions important in seed development in other species, such as AFP1-RELATED protein (*PHAVU_009G202000g*), expressed in embryos during the latest stages of seed maturation of Arabidopsis, and PPR protein (*PHAVU_009G175100g*), which play important roles in seed development in higher plants (Li et al. 2021). MAPKs control signaling cascades that play essential roles in plant growth, development, and defense response (Jiang et al. 2022), and are involved in regulating seed size in rice (Tian et al. 2021; Wu et al. 2022). The gene *PHAVU_009G062400g,* which encodes a MAP3K3/MEKK3, could be part of this network. Acting upstream of the MAPK gene in Arabidopsis can be found LecRK-VIII.2 that coordinates silique number, seed size, and seed number to determine seed yield (Xiao et al. 2021). A homologous of this gene is a DEG located under the QTL Seed08_55.3, *PHAVU_008G239600g* which encodes a LECTIN RECEPTOR KINASE VIII.1.

Four bean genomes with predicted genes are available: one from the Andean gene pool (G19833) and three closely related to the Mesoamerican gene pool (UI111, Labor Ovalle, and 5-593). Genotype G19833 has seeds larger than the other three genotypes. The 22 DEGs located in the consistent QTL associated with SW showed high variation when the respective sequences were aligned with the genes predicted in G19833, which may be a consequence of evolutionary differentiation and may contribute to phenotypic differentiation for SW. This variation was not homogeneous among the three MA genotypes, and 12 predicted genes in genotype 5-593 were very similar to those in G19833, suggesting that this observed variation should not be relevant to explicating the phenotypic variation between both gene pools for SW. In contrast, 10 genes were highly variable to those of G19833 in the three MA genotypes (see Table S8), suggesting that they could be relevant for phenotypic variation between both gene pools for seed weight. Interestingly three of them (*PHAVU_001G267600g, PHAVU_001G268900g, PHAVU_010G123100g*) were located in the QTLs that provided the greatest differences in SW: Seed01_51.9 and Seed10_39.1. From the sequences of these genes, functional markers tagging both genes and QTL regions can be developed and used in marker-assisted selection in breeding programs where SW is a trait considered. However, evidence suggests that SW is not the result of variation in a few loci; rather, it is the consequence of variation in many loci and their interactions with the environment in which the plants develop. Thus, these markers can help to enrich segregating populations in certain SW phenotypes.

Finally, the combination of GWAS and RNA-seq analyses helped elucidate QTL regions and candidate genes that control seed size, shape, and quality traits. GWAS revealed 23 QTL regions that were significantly associated with the evaluated traits, 13 of which were consistent with the regions reported in previous studies. These QTL regions contained 1,605 annotated genes in the G19833 bean genome, of which 91 genes were differentially expressed during seed development in the cultivar ‘Xana.’ DEGs were only found in 16 QTL, and 22 DEGs were located in five consistent QTL regions associated with SW. These regions and DEGs constitute a priority set for future genetic studies focused on SW control, their identification increases our knowledge of the genetic architecture of this trait, and a marker can be use as indirect selection tool, which is a relevant characteristic in many breeding programs.

### Supplementary Information

Below is the link to the electronic supplementary material.

Supplementary file1 (DOCX 3057 kb)

Supplementary file2 (XLSX 4587 kb)

Supplementary file3 (XLSX 34 kb)

Supplementary file4 (XLSX 3009 kb)

Supplementary file5 (XLSX 200 kb)

Supplementary file6 (XLSX 17 kb)

Supplementary file7 (XLSX 45 kb)

Supplementary file8 (XLSX 16 kb)

Supplementary file9 (XLSX 13 kb)

## Data Availability

The authors confirm that the data supporting the findings of this study are available within the article [and/or] its supplementary materials. An image of the seeds of each line included in the Spanish Diversity Panel (SDP) was deposited in the Zenodo repository (https://zenodo.org/records/10263706).
