# Supplementary material for: Identification of consistent QTL and candidate genes associated with seed traits in common bean by combining GWAS and RNA-Seq
Source: Theor Appl Genet. 2024 May 27;137(6):143. doi: 10.1007/s00122-024-04638-5 (PMC11130024; doi:10.1007/s00122-024-04638-5)
Supplement: Supplementary file 1 — Supplementary file1 (DOCX 3057 kb) [file 122_2024_4638_MOESM1_ESM.docx]

**Figures extra**

**
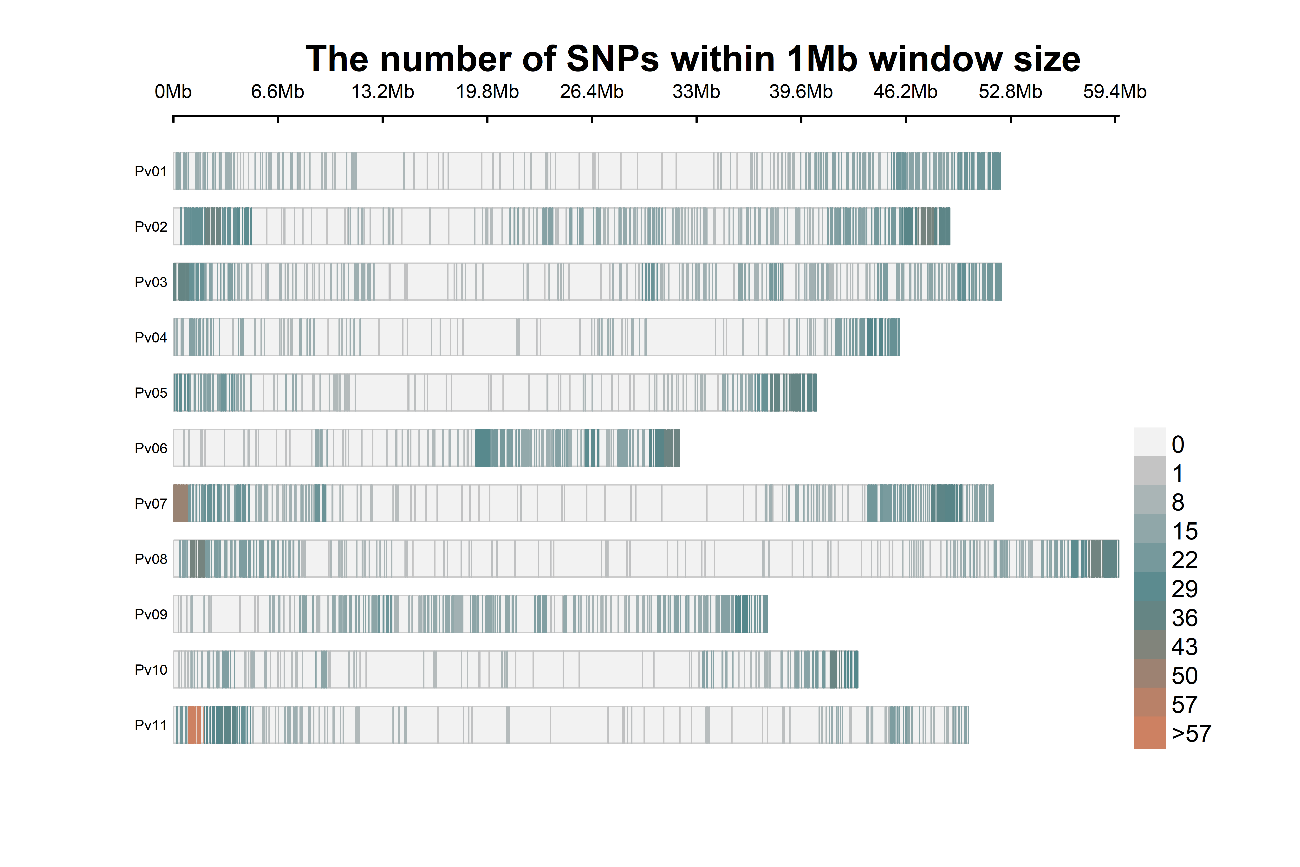
**

**Figure S1.** Distribution of SNPs across 11 bean chromosomes.


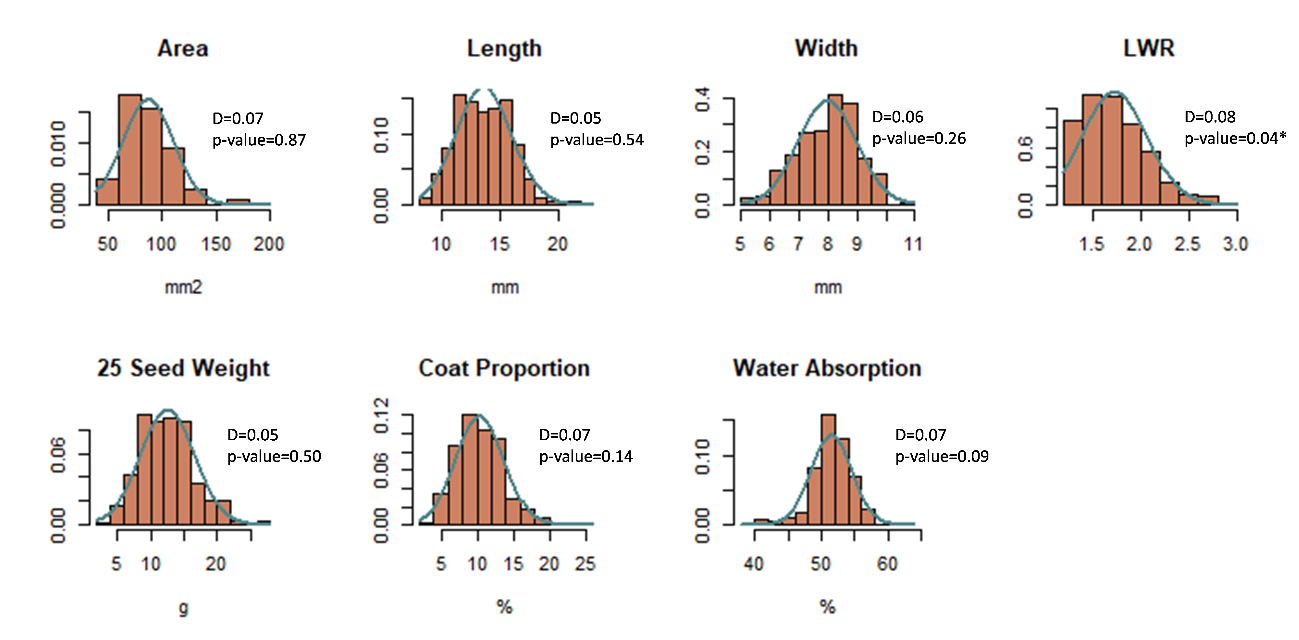


**Figure S2.** Phenotypic frequency distribution of adjusted means of the seven seed traits evaluated in the Spanish Diversity Panel.


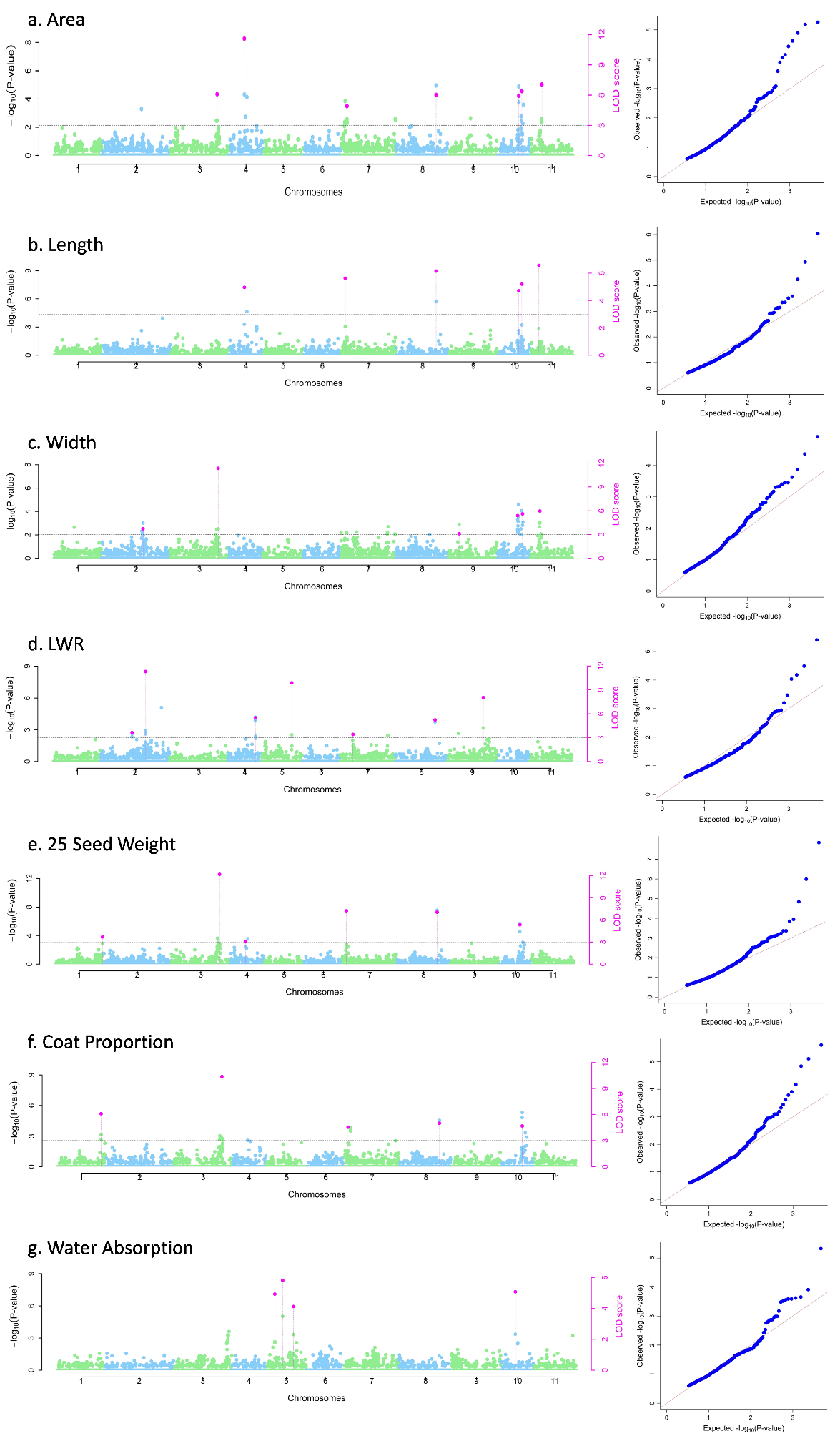


**Figure S3.** Manhattan and QQ plots were obtained using the FASTmrEMMA method for morpho-agronomic traits. a) area, b) length, c) width, d) LWR, e) Seed Weight, f) coat proportion, and g) water absorption.


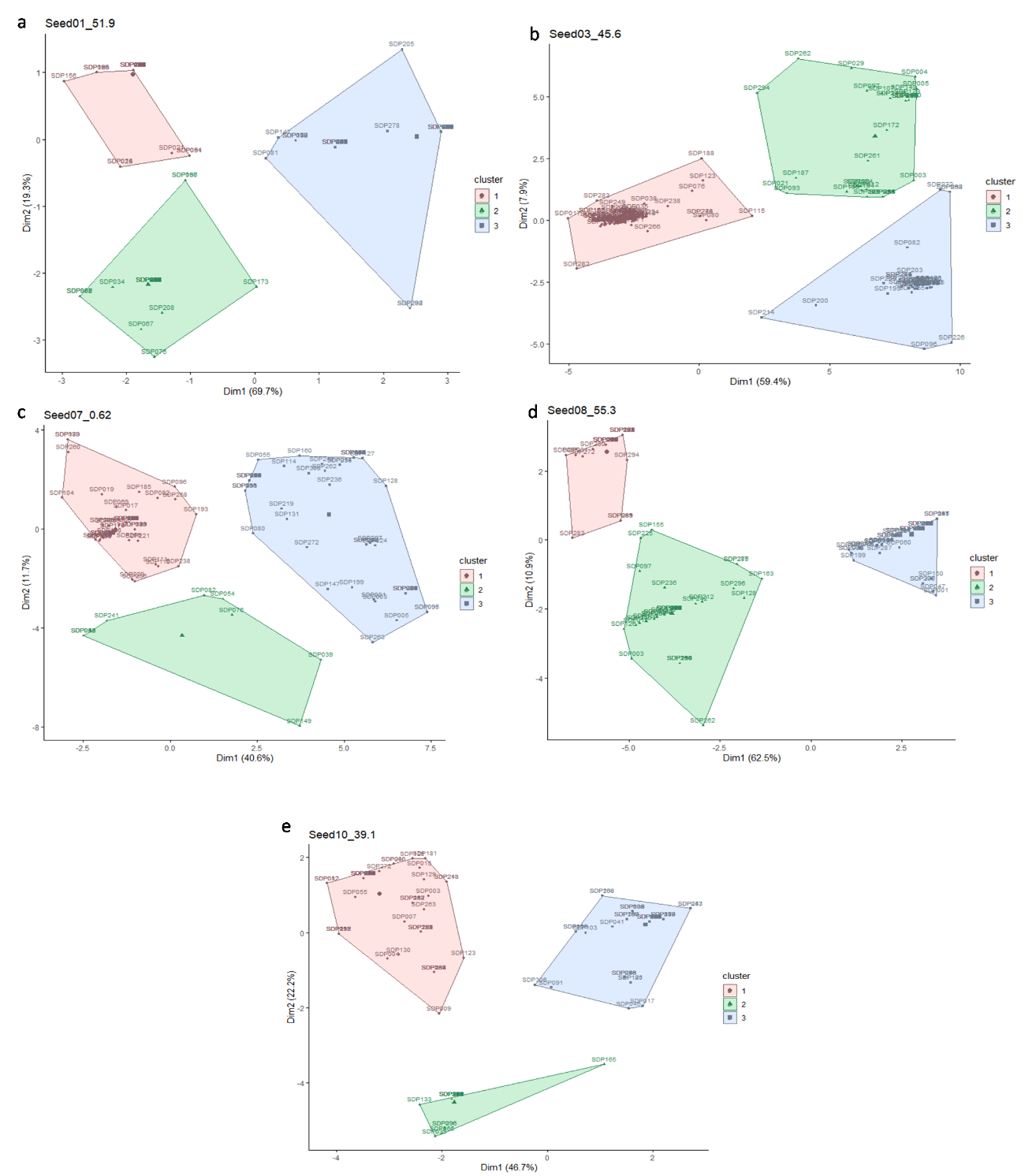


**Figure S4.** HCPC SNP data of the six regions associated with SW. a) Seed01_51.9. b) Seed03_45.6. c) Seed07_0.62. d) Seed08_55.3. e) Seed10_39.1.


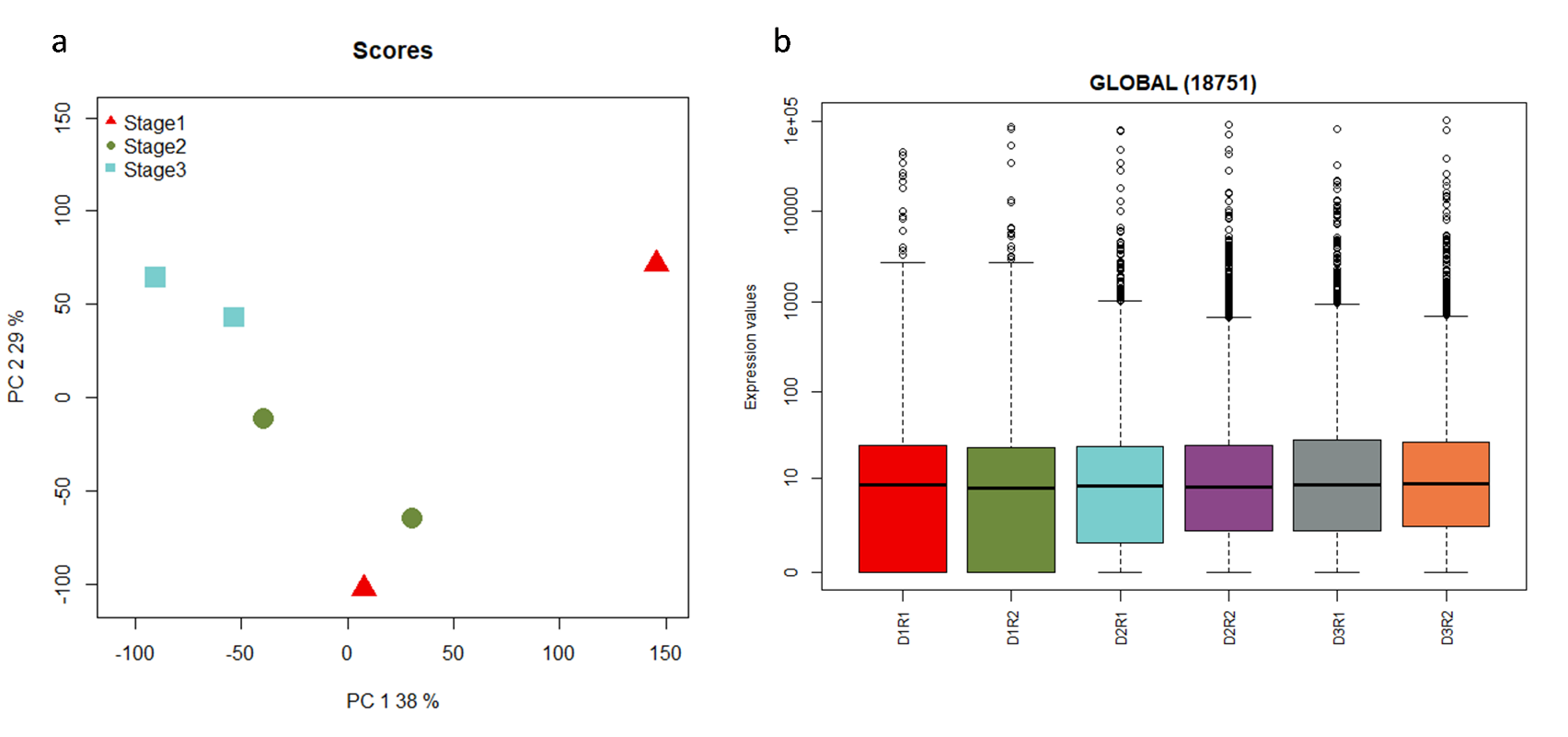


**Figure S5.** RNA-Seq data quality a) Scatterplots of the two principal components over the TMM-normalized data contain all samples. b) Boxplots with TMM-normalized data contain all loci after normalization.


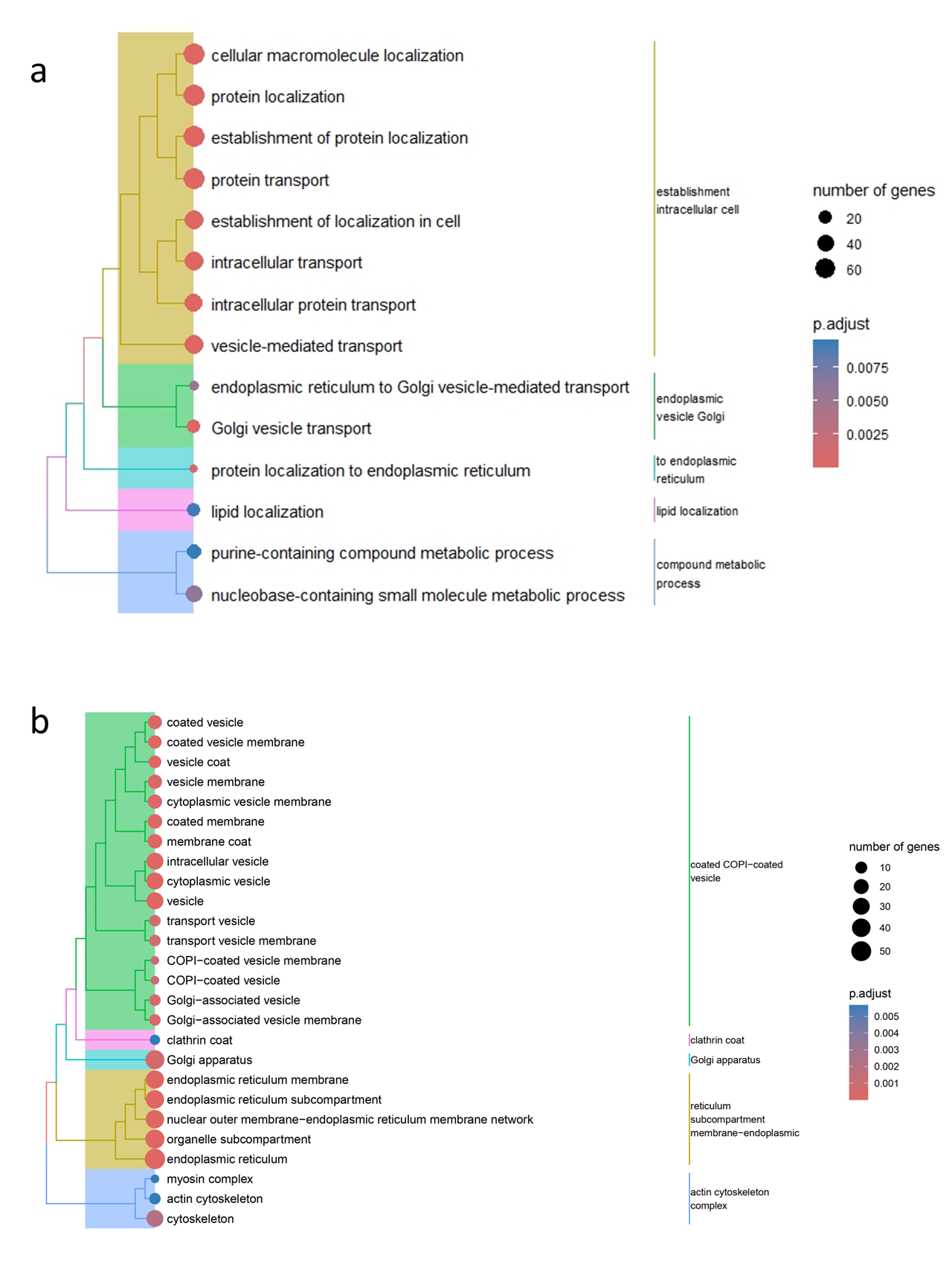


**Figure S6.** GO term enrichment for the DEGs found in the comparison of D3 vs. D1. a) GO terms in the Biological Process (BP) category. b) GO terms for Cellular Components (CC).
